# Supplementary material for: Physico-Chemical and Antimicrobial Efficacy of Encapsulated Dhavana Oil: Evaluation of Release and Stability Profile from Base Matrices
Source: Molecules. 2022 Nov 8;27(22):7679. doi: 10.3390/molecules27227679 (PMC9693536; doi:10.3390/molecules27227679)
Supplement: Supplementary file 1 [file molecules-27-07679-s001.zip › molecules-1999371-supplementary.pdf]

Physico-chemical and release profile of Encapsulated Dhavana oil from base powder matrices and their antimicrobial efficiency

Shirish K. Phanse[1], Shriya Sawant[2], Harinder Singh[2] and Sudeshna Chandra[1*]

[1]Department of Chemistry
[2]Department of Biological Sciences
Sunandan Divatia School of Science, SVKM's NMIMS (Deemed to be) University
V. L. Mehta Road, Vile Parle (West), Mumbai-400056 India

SUPPORTING INFORMATION

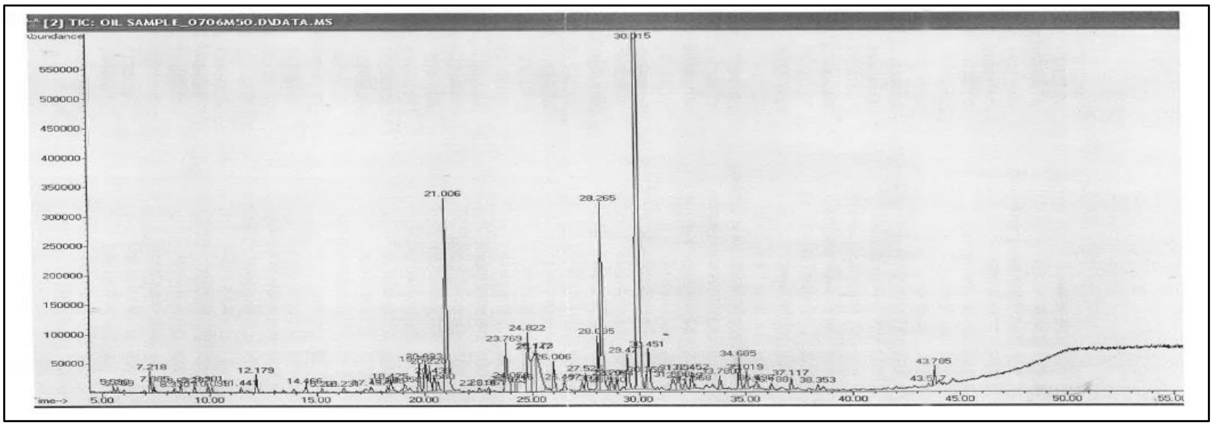

GCMS of original Dhavana oil from Leela Aromatics ltd.

PK	RT	Area Pct	Library/ID	Ref	CAS	Qual
1	5.5304	0.0985	Ethyl-2-Methyl Butyrate (fema 2443)	16412	007452-79-1	90
2	5.7089	0.0809	ISOVALERATE ETHYLE	12131	000108-64-5	78
3	7.2183	0.3927	pic A de less. davana	3025	000000-00-0	70
4	7.385	0.1089	isovalerate de propyle	3017	000000-00-0	91
5	8.3299	0.0624	gamma-terpinene	10969	000099-85-4	96
6	8.6319	0.2065	PARA CYMENE	12774	000099-87-6	94
7	9.2872	0.1295	pic B de less. davana	3026	000000-00-0	91
8	9.901	0.136	2-methylbutyrate de 2-methylbutyle	252	000000-00-0	90
9	10.0991	0.0667	isoAmyl isoValerate (fema 2085)	16586	000659-70-1	90
10	11.4409	0.1029	4-Thujanol (fema 3239)	16620	000546-79-2	90
11	12.1786	0.3547	linalol	1165	000000-00-0	91
12	14.4659	0.2283	1-terpinene-4-ol = 4-terpineol	1354	000562-74-3	96
13	15.2208	0.1018	dimethyl furanone	11648	000000-00-0	78
14	16.2306	0.1235	beta-elemene	442	000000-00-0	87
15	17.4968	0.1876	2-oxo-5-methyl-5-vinyl THF	11649	001073-11-6	49
16	18.2652	0.1252	nor-davanone : pic 1	3018	000000-00-0	59
17	18.4247	0.3364	ALLOAROMADENDRENE	13278	025246-27-9	96
18	19.0504	0.2599	ledene = viridiflorene	2897	000000-00-0	91
19	19.7368	0.7126	acetate de geranyle	8942	000105-87-3	91
20	20.0233	1.1004	SELINENE BETA	13100	017066-67-0	99
21	20.2203	0.9105	GERMACRENE D	13403	023986-74-5	91
22	20.4393	0.4599	davana furane : pic 1	3019	000000-00-0	93
23	20.5802	0.3735	clovene (Acedesa) : pic 2	2098	000000-00-0	72
24	21.0056	6.8708	bicyclogermacrene	7469	000000-00-0	98
25	22.516	0.1353	phenylpropionate dethyle	2096	000000-00-0	93
26	22.9615	0.1283	methyl cinnamate	10892	000103-26-4	97
27	23.7686	1.8005	davana ether ? (tres probable) : pic 1	3023	000000-00-0	81
28	23.9711	0.0624	2-mercaptopyridine = 2-pyridinethiol	2157	000000-00-0	10
29	24.0595	0.7285	2-ethoxyanisole = ethyl methyl pyrocatechol	1729	000000-00-0	46
30	24.2464	0.272	tabanone (Dragoco) : pic 4 (10%)	10377	000000-00-0	9
31	24.8221	2.6305	ethyl cinnamate	10795	000103-36-6	98

GCMS of original Dhavana oil from Leela Aromatics ltd.

31	24.8221	2.6305	ethyl cinnamate	10795	000103-36-6	98
32	25.1469	2.123	davana ether ? (tres probable) : pic 1	3023	000000-00-0	32
33	25.1726	3.0251	(E)-cinnamate de dihydrolinalyle	3966	000000-00-0	32
34	26.0056	0.9176	CINNAMATE METHYLE TRANS (2/2)	12240	000103-26-4	97
35	26.4973	0.2991	(E)-nerolidol = trans-nerolidol	974	007212-44-4	91
36	27.333	0.2778	artedouglasia oxyde = davana dihydropyrone : pic 1	3020	000000-00-0	87
37	27.5247	0.5487	dovanone	10935	000000-00-0	93
38	28.0855	1.4919	dovanone	10935	000000-00-0	93
39	28.2653	5.6005	Ethyl Cinnamate (fema 2430)	16424	000103-36-6	98
40	28.5899	0.1811	beta-patchoulene	1343	000000-00-0	30
41	28.704	0.4939	2-butyl-2-pentenal = 2-propylidenehexanal	8677	000000-00-0	43
42	28.9539	0.3602	davanone : pic 1	3021	000000-00-0	95
43	29.4707	1.2507	spathulenol	2304	006750-60-3	95

GCMS of original Dhavana oil from Leela Aromatics ltd.

44	30.0149	56.3217	dovanone	10935	000000-00-0	98
45	30.3505	0.3624	MASSOIA LACTONE PROPYL	12819	000000-00-0	47
46	30.4513	1.49	T-cadinol	2972	000000-00-0	91
47	31.5882	0.6109	alcool sesquiterpenique A de less. marjolaine	4707	000000-00-0	96
48	31.8341	0.7286	trans-pinocamphone	814	000000-00-0	35
49	32.1493	0.3556	pic C de less. davana	3027	000000-00-0	91
50	32.452	0.5393	beta-eudesmol	174	000000-00-0	98
51	32.5675	0.125	6-methyl-2-methylthiopyrazine	2060	000000-00-0	11
52	33.7805	0.455	(E)-tagetone = trans-tagetone	2525	000000-00-0	50
53	34.6851	0.8811	davana ether monocyclique : pic 1	3022	000000-00-0	98
54	35.0194	0.5455	davana ether monocyclique : pic 1	3022	000000-00-0	68
55	35.4969	0.2366	davanone : pic 1	3021	000000-00-0	32
56	35.5359	0.1748	rhubofix (Firm.) : pic 1	1578	000000-00-0	11
57	36.1878	0.2159	pic L du resin. alc. benjoin Sumatra (3 isomeres visibles)	8649	000000-00-0	52
58	37.1174	0.3629	methyl jasmonate 1/2	11081	039924-52-2	99
59	38.3526	0.1881	pic C de labs. jasmin sambac Malaisie (oplopanone ?)	5628	000000-00-0	27
60	43.5172	0.0961	10-Mercaptopinane (fema 3503)	16214	023832-18-0	30
61	43.7847	0.4528	hydroxydavanone	3024	000000-00-0	87

Figure S1: GC-MS analysis of neat Dhavana oil and its components.

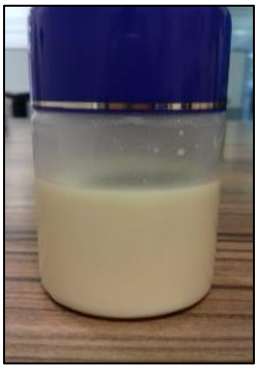

Figure S2: Stable Emulsion of a Dhavana oil dispersed in Capsule^{TM} gum after 7 days

**Sr.no.**	**Scale of Olfactive Intensity**	**Score**
1	No odour	1
2	Threshold Odour	2
3	Very Slight Odour	3
4	Slight Odour	4
5	Slight to moderate Odour	5
6	Moderate Odour	6
7	Slightly Strong Odour	7
8	Moderately Strong Odour	8
9	Strong Odour	9
10	Very Strong Odour	10

Table S1: Scale of Olfactive Intensity

1. Thermal Degradation pattern of neat Dhavana oil & Encapsulated Dhavana oil in two different powder bases TALC & $CaCO_3$.The sample were prepared as below.
    a. B. no 001 - Neat Dhavana oil in TALC.
    b. B.no 002 - Encapsulated Dhavana oil in TALC.
    c. B.no 003 - Neat Dhavana oil in $CaCO_3$.
    d. B.no 004 - Encapsulated Dhavana oil in $CaCO_3$.
    e. B.no 005 - Dhavana oil in Starch please rectify.

Figure- S3

Neat Dhavana oil in TALC

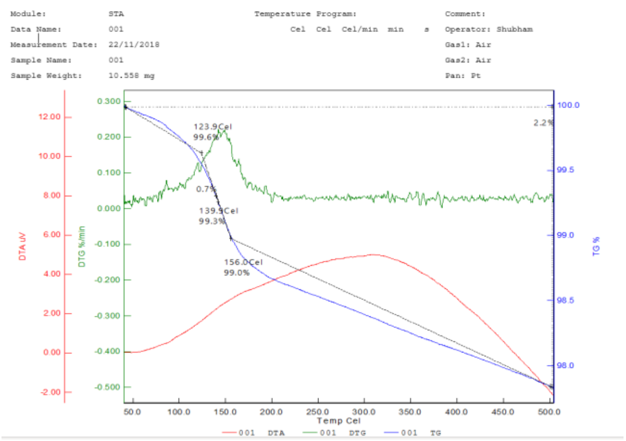

Encapsulated Dhavana oil in TALC.

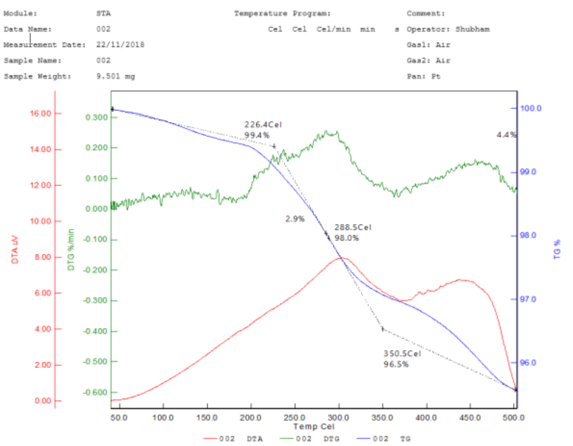

Neat Dhavana oil in $CaCO_3$

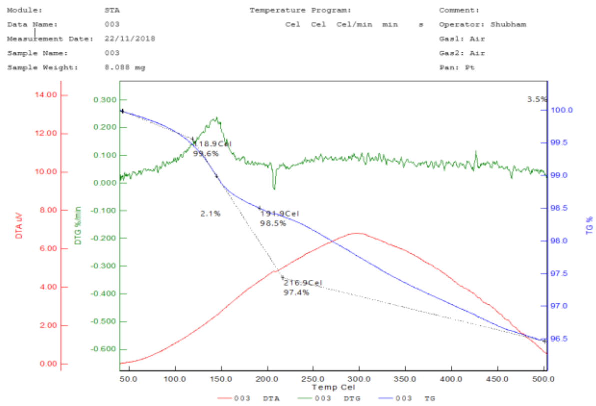

Encapsulated Dhavana oil in $CaCO_3$

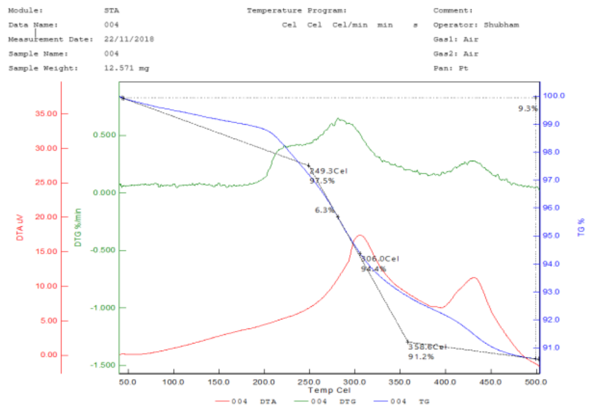

.

**Name of sample**	**Fridge 3^{0}C**	**22 ℃ (RT)**	**45^{0}C**	**37^{0}C&70% RH**	**Time period**
Dhavana oil in TALC-B.no-001	Yes	Yes	Yes	Yes	1M/2M/3M
Encap.Dhavana oil in TALC-B.no - 002	Yes	Yes	Yes	Yes	1M/2M/3M
Dhavana oil in $CaCO_3$. B.no -003	Yes	Yes	Yes	Yes	1M/2M/3M
Encap. Dhavana oil in $CaCO_3$ B.no-004	Yes	Yes	Yes	Yes	1M/2M/3M

Table S2: Experimental conditions for evaluating stability and olfactory profile

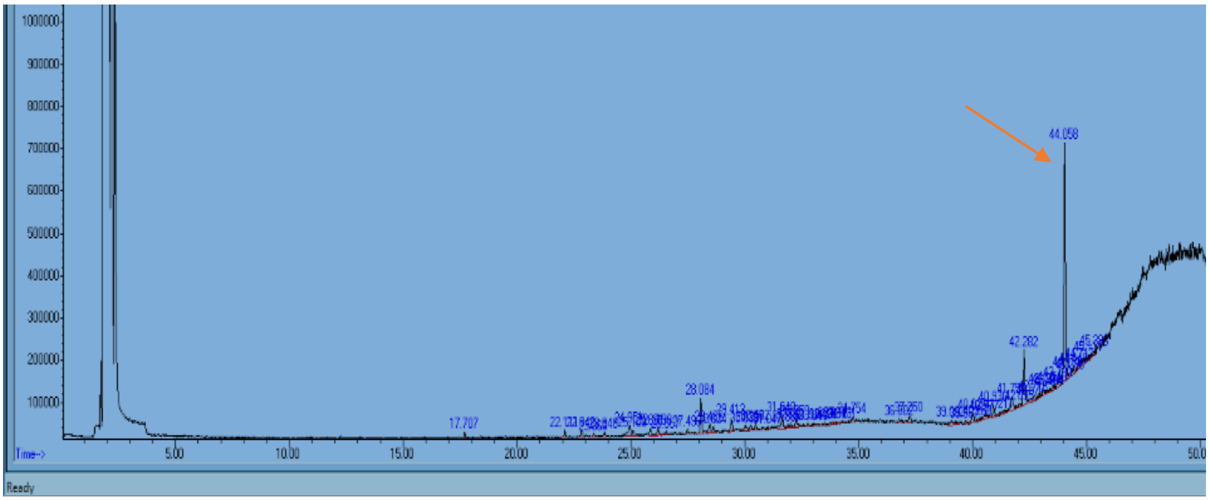

Figure S4a: GCMS profile of neat Dhavana oil in TALC after one-month storage at 45 ℃, showing the presence of hydroxydhavanone at RT-44.0583.

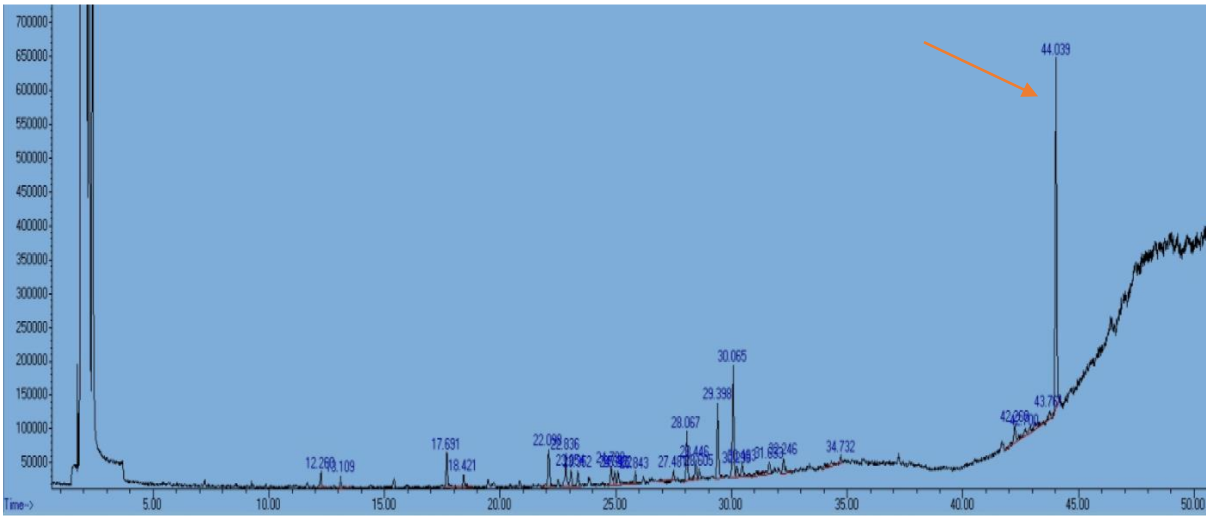

Figure S4b: GCMS profile of encapsulated Dhavana oil in TALC after one month storage at 45 ℃, showing the presence of hydroxydhavanone at RT-44.0395.

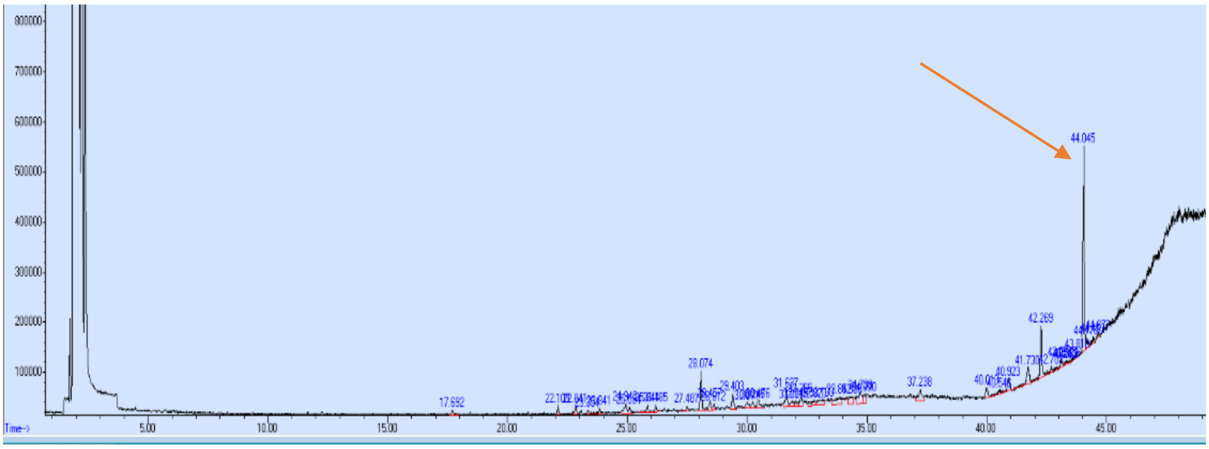

Figure S5a: GCMS profile of neat Dhavana oil in TALC after two-month storage at 45 °C, showing the presence of hydroxydhavanone at RT@44.0453.

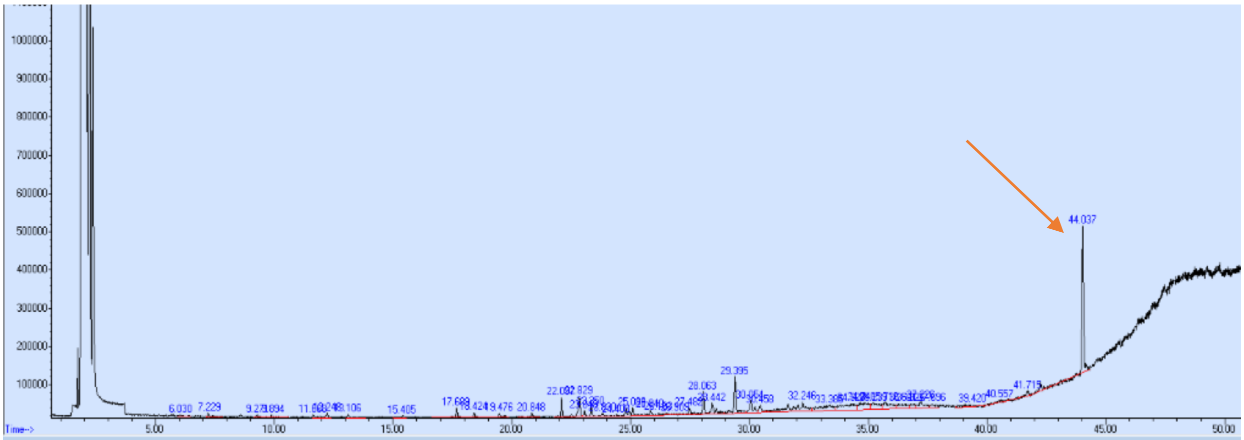

Figure S5b: GCMS profile of encapsulated Dhavana oil in TALC after two-month storage at 45 °C, showing the presence of hydroxydhavanone at RT-44.0364.

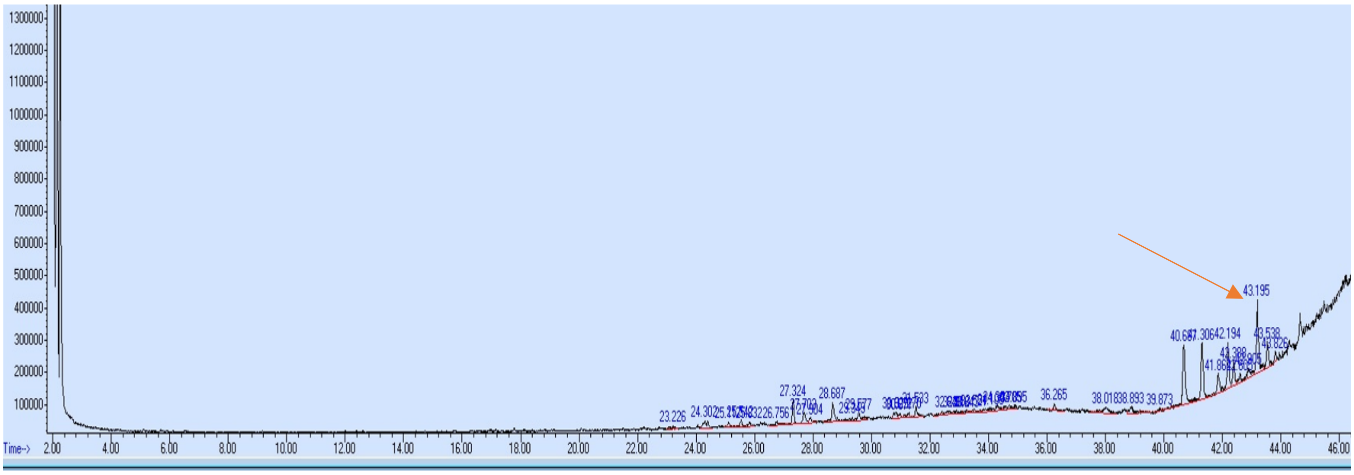

Figure S6a: GCMS profile of Dhavana oil in $CaCO_3$ *after one month at 45 °C, showing the presence of hydroxydhavanone @* RT-43.195 @Area pact – 10.600

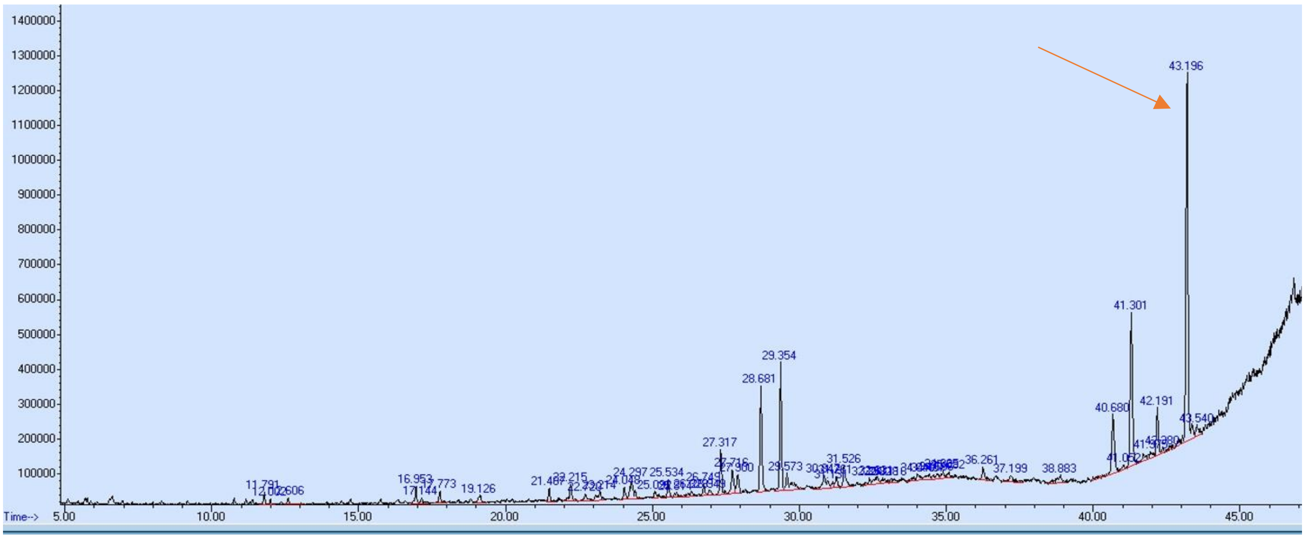

Figure S6b: GCMS profile of Encapsulated Dhavana oil in $CaCO_3$ *after one month at 45 °C, showing the presence of hydroxydhavanone @* RT- 43.196 @Area pact – 25.00

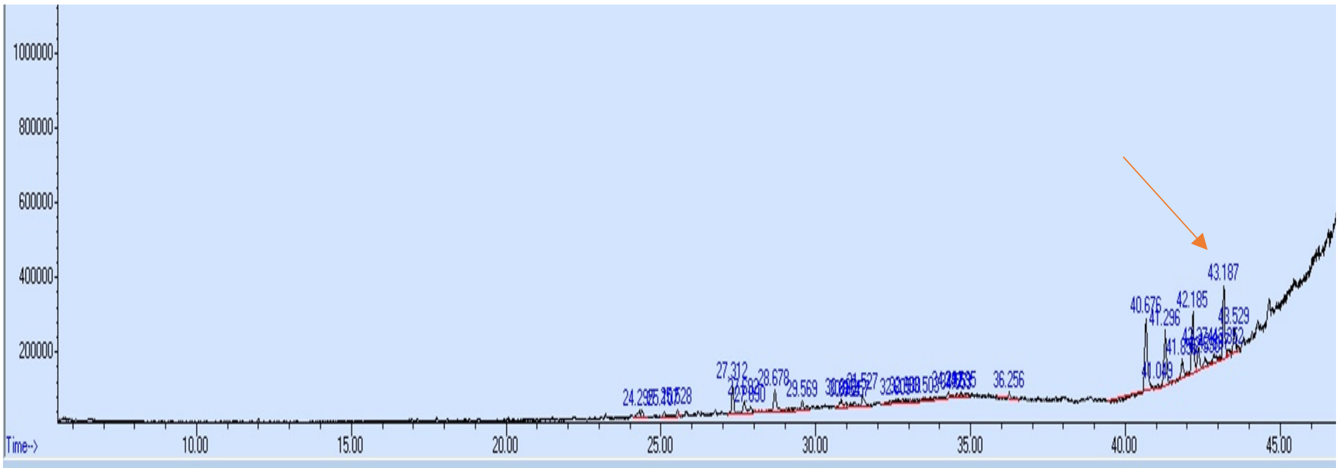

Figure S7a: *GCMS profile of Dhavana oil in* $CaCO_3$ *after two months at 45* °C, *showing the presence of hydroxydhavanone @* RT-43.187 @Area pact –10.710

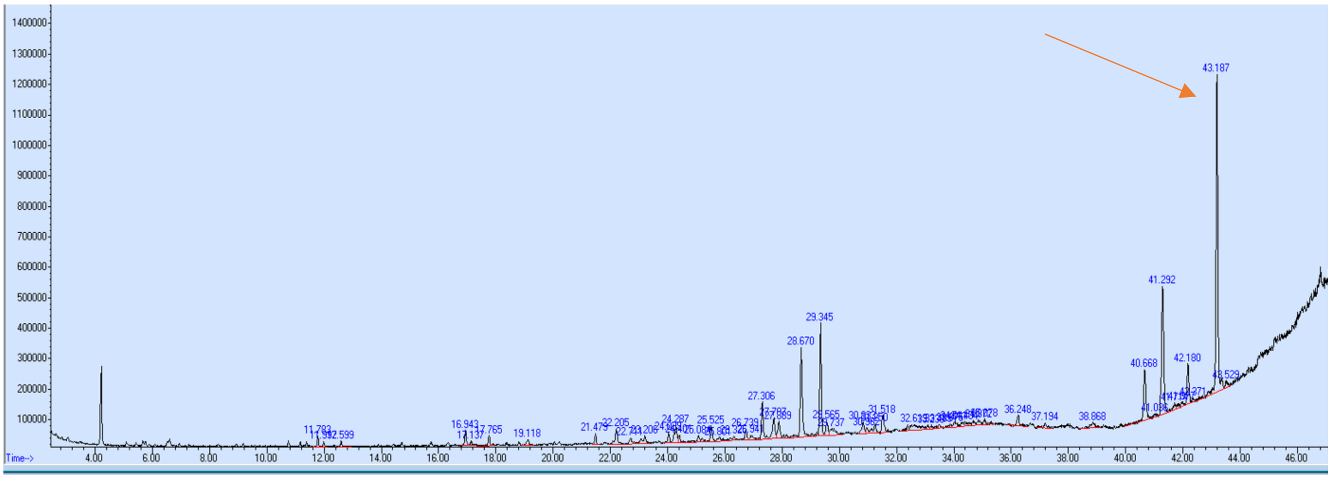

Figure S7b: GCMS profile of Encapsulated Dhavana oil in $CaCO_3$ *after two months at 45* °C, *showing the presence of hydroxydhavanone @* RT-43.187 @Area pact –24.52

Release study of neat and encapsulated Dhavana oil from TALC and $CaCO_3$ were carried by exposing the samples for 1 week in open condition (Petri dish) at RT (22 °C) and 45 °C.

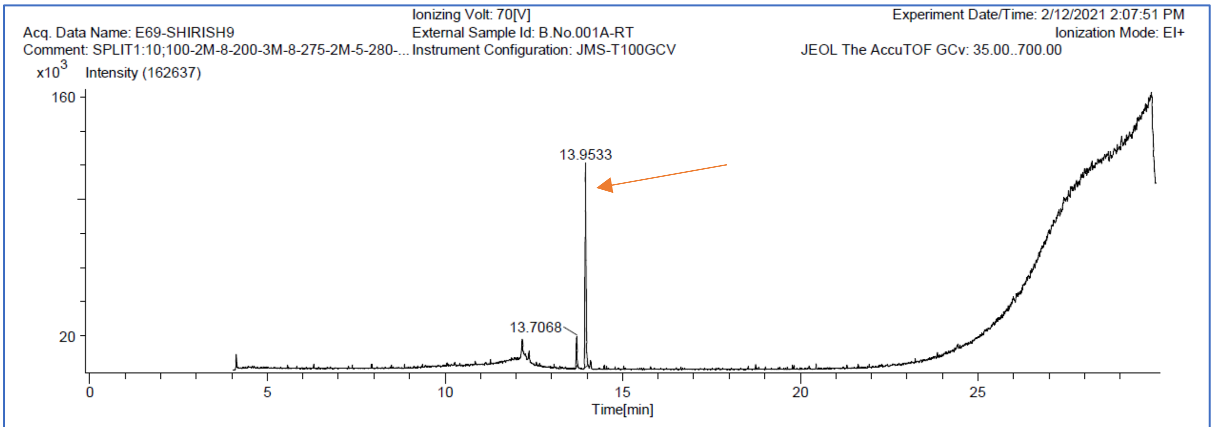

Peak Number	Time [min]	Type	Peak Width(FWH [min]	Area [Intens. * sec]	Height	Description	Start Point Time[min]	Start Point Height	End Point Time[min]	End Point Height
1	13.7068	BB	0.0278	29153.97	17254.19		13.6602	586	13.7334	3353
2	13.9533	BB	0.0316	239954.19	114171.07		13.8866	406	14.0265	2864

Figure S8a: B.no 001A – Neat Dhavana oil in TALC for RT (22 °C) / 1 Week-open petri dish

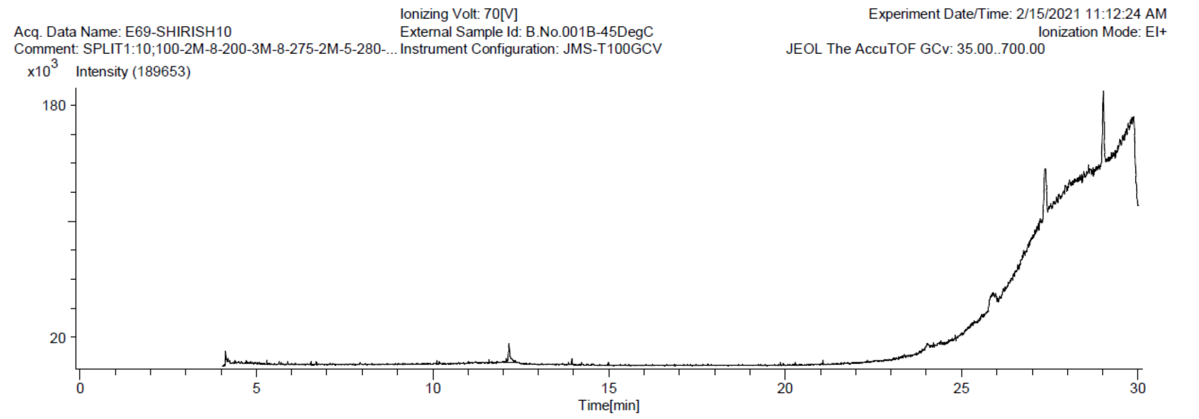

Peak Number	Time [min]	Type	Peak Width(FWH [min]	Area [Intens. * sec]	Height	Description	Start Point Time[min]	Start Point Height	End Point Time[min]	End Point Height
1	12.1622	BB	0.0399	50760.81	12679.94		12.0356	2536	12.3687	2706
2	13.9474	BB	0.0272	7198.18	4123.82		13.8941	788	13.9874	910

Figure S8b: B.no 001B – Neat Dhavana oil in TALC for 45 °C / 1 Week-open Petri dish. Active Hydroxydavanone not detected.

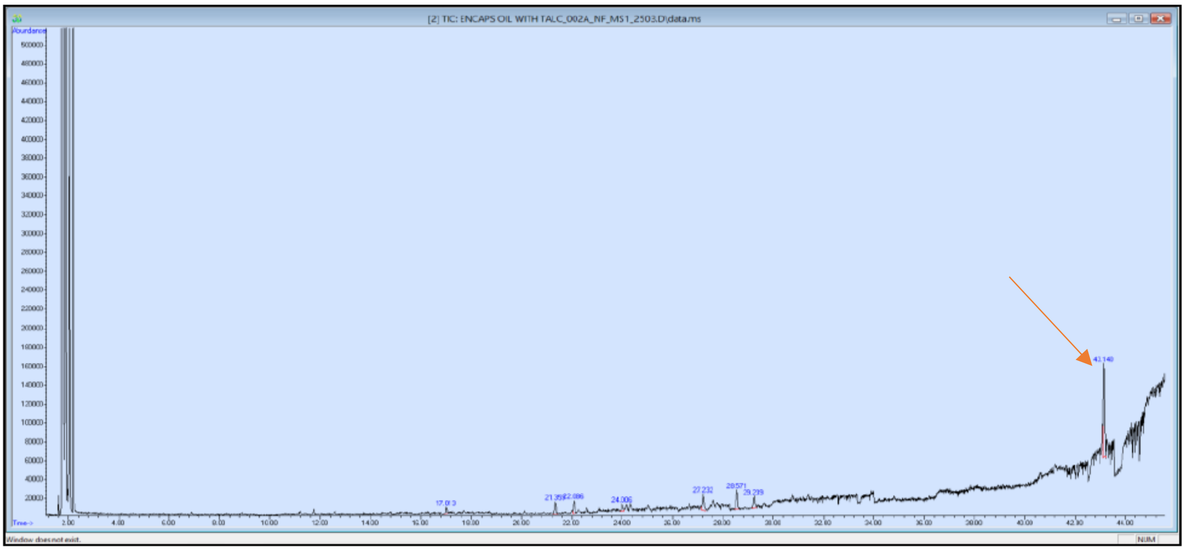

Figure S9 a: B.no 002 A – Encapsulated Dhavana oil in TALC for RT (22 °C) / 1 Week – open petri dish

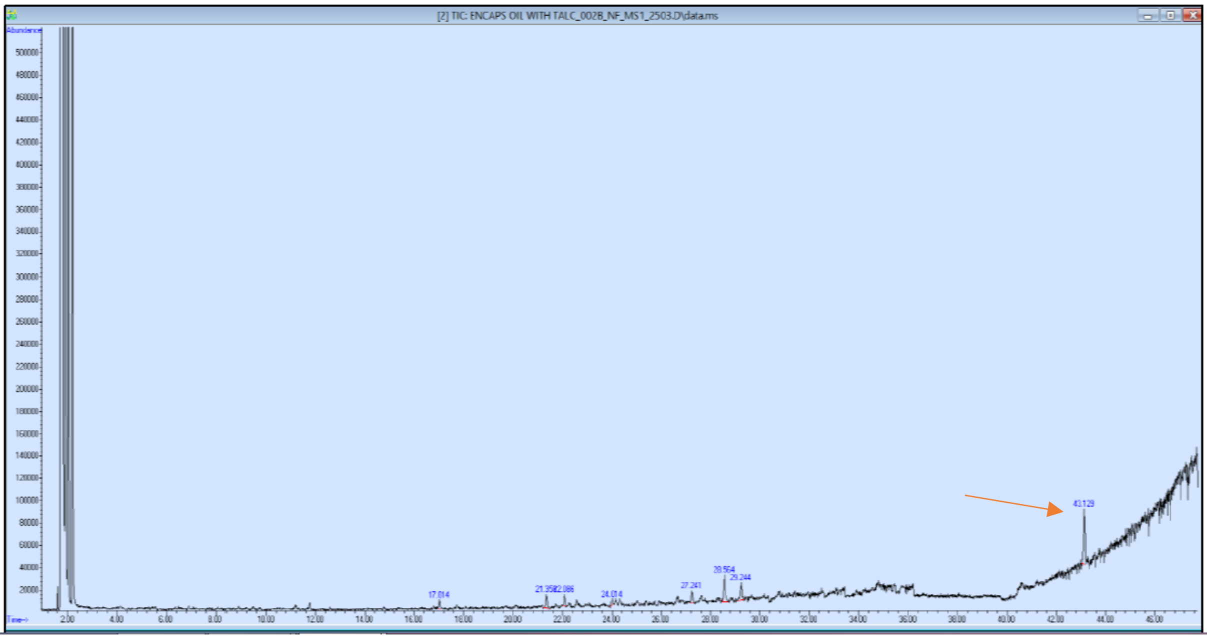

Figure S9b: B.no 002B – Encapsulated Dhavana oil in TALC for 45°C /1 Week– open petri dish.

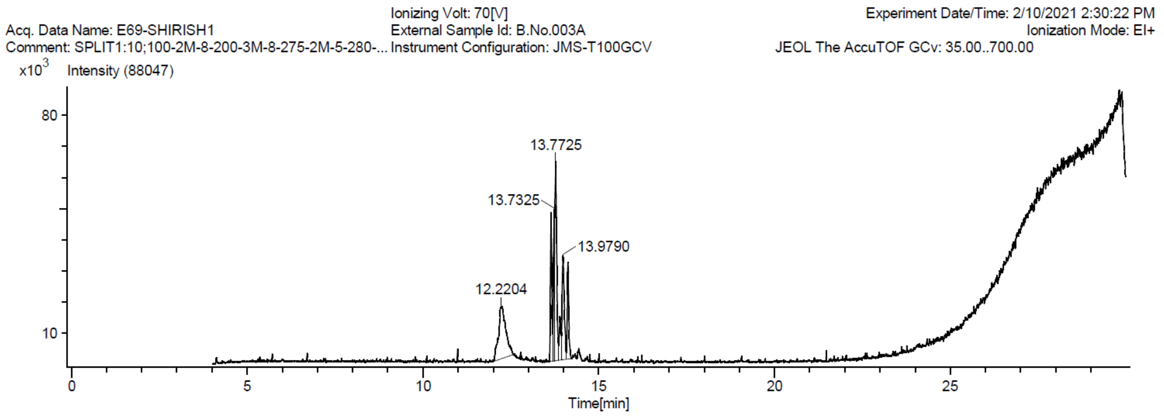

Peak Number	Time [min]	Type	Peak Width(FWH [min]	Area [Intens. * sec]	Height	Description	Start Point Time[min]	Start Point Height	End Point Time[min]	End Point Height
1	12.2204	BB	0.2093	244913.57	16670.75		12.0006	843	12.5934	3200
2	13.6393	BV	0.0438	135086.51	47901.89		13.5793	786	13.6925	962
3	13.7325	VV	0.0387	112959.27	48712.48		13.6925	962	13.7458	1045
4	13.7725	VV	0.0586	227017.69	63839.90		13.7458	1045	13.8591	1221
5	13.8990	VV	0.0596	43527.22	13961.26		13.8591	1221	13.9323	1335
6	13.9790	VV	0.0684	146842.27	33443.44		13.9323	1335	14.0789	1563
7	14.1255	VB	0.0459	95543.39	31026.07		14.0789	1563	14.2321	1801

Figure S10a: B.no 003A – Neat Dhavana oil in $CaCO_3$ for RT (22 ℃) / 1 Week– open petri dish

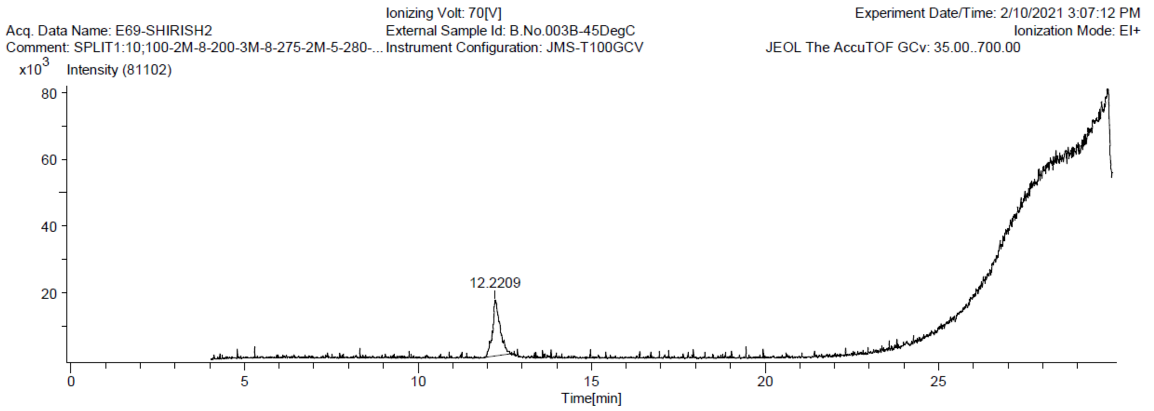

Peak Number	Time [min]	Type	Peak Width(FWH [min]	Area [Intens. * sec]	Height	Description	Start Point Time[min]	Start Point Height	End Point Time[min]	End Point Height
1	12.2209	BB	0.2033	249776.57	16809.78		11.8945	606	12.7605	1869

Figure S10b: B.no 003B – Neat Dhavana oil in $CaCO_3$ for 45 ℃ / 1 Week– open petri dish Active was not detected.

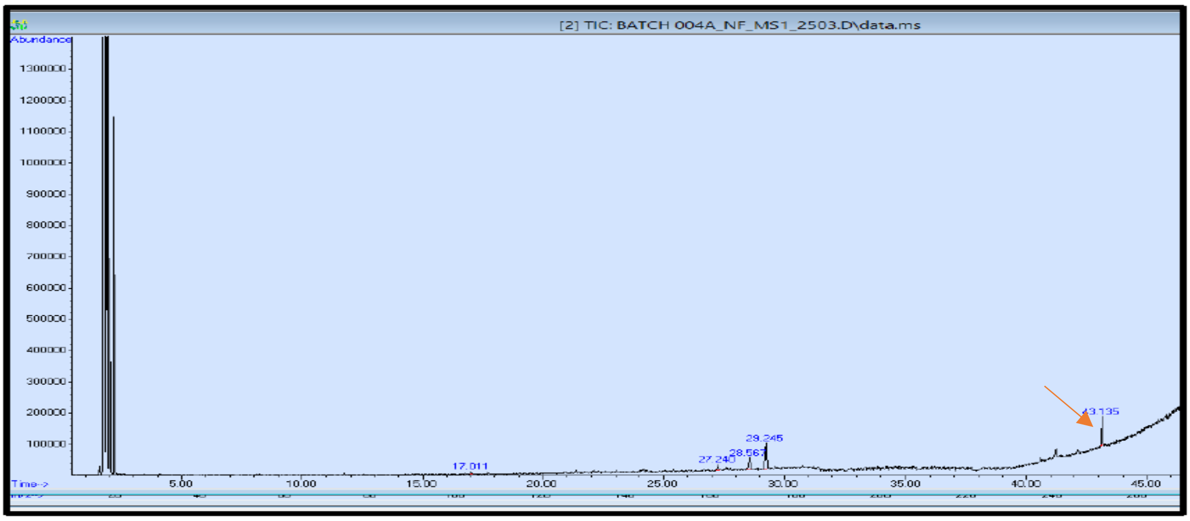

Figure S11a: B.no 004A – Encapsulated Dhavana oil in $CaCO_3$ for RT (22 ℃) / 1 Week-open petri dish

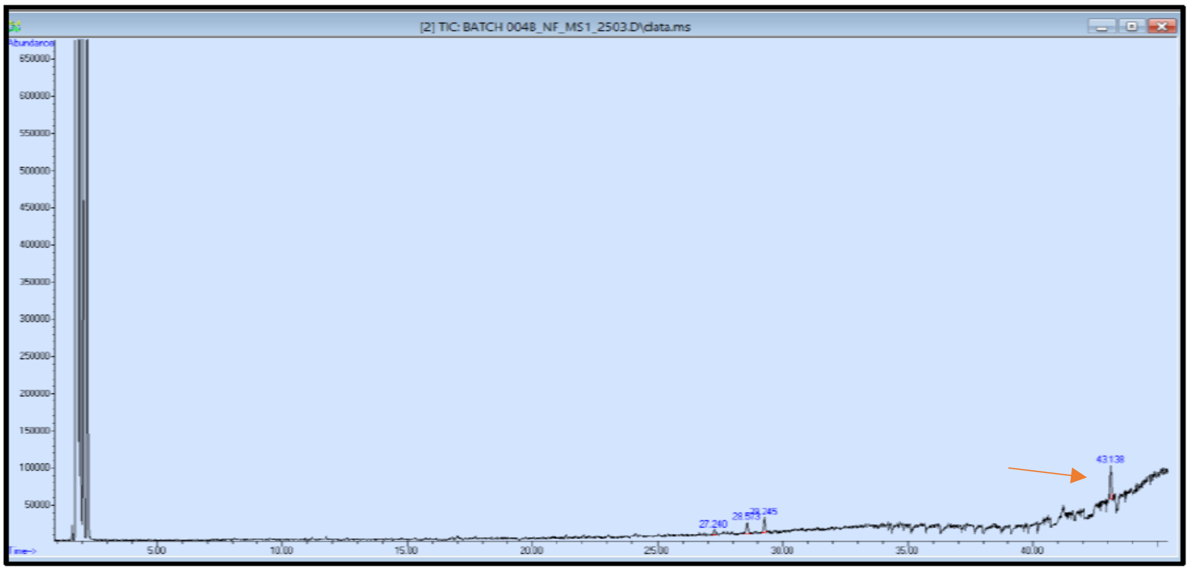

Figure S11b: B.no 004B – Encapsulated Dhavana oil in $CaCO_3$ for 45 ℃ / 1 Week-open petri dish

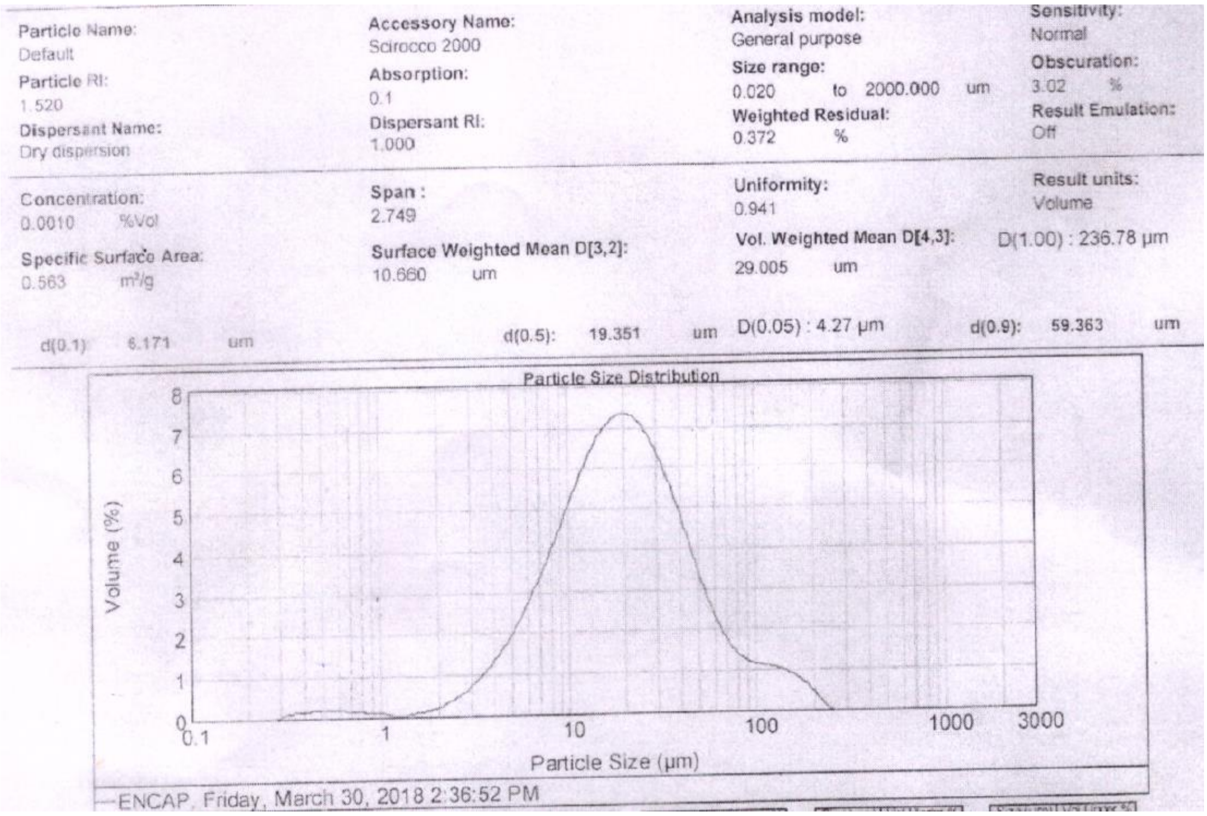

Figure S12: Particle size distribution of Encapsulated Dhavana oil.
